# A BRCA2 germline mutation and high expression of immune checkpoints in a TNBC patient

**DOI:** 10.1038/s41420-023-01651-3

**Published:** 2023-10-09

**Authors:** Yuyi Han, Valentina Rovella, Artem Smirnov, Oreste Claudio Buonomo, Alessandro Mauriello, Tommaso Perretta, Yufang Shi, Jonathan Woodmsith, Julia Bischof, TOR CENTRE, Pierluigi Bove, Hartmut Juhl, Manuel Scimeca, Giuseppe Sica, Giuseppe Tisone, Ying Wang, Erica Giacobbi, Marco Materazzo, Gerry Melino, Eleonora Candi, Francesca Bernassola

**Affiliations:** 1 Department of Experimental Medicine, TOR, University of Rome Tor Vergata, 00133 Rome, Italy; 2 Department of Ophthalmology, The Affiliated Hospital of Jiangnan University, 214000 Wuxi, China; 3 Biochemistry Laboratory, Istituto Dermopatico Immacolata (IDI-IRCCS), 00100 Rome, Italy; 4 Department of Diagnostic Imaging and Interventional Radiology, Policlinico Tor Vergata University, Rome, 00133 Italy; 5 The Third Affiliated Hospital of Soochow University, Institutes for Translational Medicine, Soochow University, Suzhou, 215000 China; 6 Indivumed GmbH, Falkenried, 88 Building D, 20251 Hamburg, Germany; 7 Shanghai Institute of Nutrition and Health, Shanghai, 200031 China; 8 Department of Experimental Medicine, Policlinico Tor Vergata University, Rome, 00133 Italy; 9 Department of Surgical Science, Policlinico Tor Vergata University, Rome, Italy

**Keywords:** Breast cancer, Prognostic markers

## Abstract

Triple-negative breast cancer (TNBC) is the most aggressive subtype of mammary carcinoma. Here, we describe a case of an 81-year-old female diagnosed with ductal triple negative breast cancer with a germline pathogenic variant in BReast CAncer gene2 (*BRCA2*). Genetic testing also revealed the presence of four somatic mutations in the ephrin type-A receptor 3 (*EphA3*), *TP53*, *BRCA1*-associated protein (*BAP1*), and *MYB* genes. The *BRCA2*, *TP53,* and *BAP1* gene mutations are highly predictive of a defective homologous recombination repair system and subsequent chromosomal instability in this patient. Coherently, the patient displayed a strong homologous recombination deficiency signature and high tumor mutational burden status, which are generally associated with increased probability of immune neoantigens formation and presentation, and with tumor immunogenicity. Analysis of immune checkpoint revealed high expression of programmed cell death ligand 1 (PD-L1), programmed cell death ligand 2 (PD-L2), programmed death 1 (PD1), and cytotoxic T-lymphocyte-associated protein 4 (CTLA 4), suggesting that the patient might likely benefit from immunotherapies. Altogether, these findings support an unveiled link between *BRCA2* inactivation, HR deficiency and increased expression of immune checkpoints in TNBC. This clinical case highlights the importance of screening TNBC patients for genetic mutations and TMB biomarkers in order to predict the potential efficacy of immunotherapy.

## Introduction

Breast cancer is one of the most common malignancies in women worldwide [1]. It displays complex diversity in both molecular alterations, clinical manifestations, and pathological characteristics [2–5]. Estrogen receptor (ER), progesterone receptor (PR), and human epidermal growth factor receptor-2 (ERBB2/HER2) are considered as the molecular markers for diagnostic classification of breast cancer subtypes [6, 7]. Breast cancer with the genetic signature of ER-negative, PR-negative, and ERBB2/HER2-negative has been classified as triple-negative breast cancer (TNBC) [8], which represents the most aggressive clinical subtype with a poor prognosis. Breast cancer, and in particular TNBC, presents significant genomic defects [9–12] involving protein degradation [13–15], mutations or deregulation of the p53 family members of tumor suppressors [16–20] as well as of other transcription factors [21–23]. In addition, also defects in metabolism or in hypoxia response [24–27] seem to influence the ability of cancer cells to progress [28–31] or to respond to treatment [32, 33]. Compared with other subtypes, TNBC displays a greater tendency of recurrence with higher invasive and metastatic behavior. Due to the lack of targetable receptors (ER, PGR, and HER-2), TNBC patients cannot benefit from hormonal therapy or receptor-targeted monoclonal antibodies.

By using sequencing technologies, a series of molecular markers have been recognized as targetable genes so that individualized therapeutic regimens appear to be a promising approach to improve the survival of TNBC patients. Researchers have been committed to investigate effective neoadjuvant chemotherapy strategies at the molecular level, with a focus on genetic background of breast-cancer susceptibility genes such as *BRCA1/2* loss-of-function mutations.

*BRCA* mutations account for about 10–15% of TNBC patients [34, 35]. The deleterious variants of *BRCA1/2* genes, such as c.5558dupA (BIC: 5677insA) in *BRCA1* (ref. 36) and c.9541_9554del14 (BIC: 9769del14) in *BRCA2*, abrogate the function of encoded proteins, and confer a high risk of breast (most commonly the TNBC subtype) and ovarian cancers [37]. BRCA1 and BRCA2 are required for mammalian development, and they function as tumor suppressors to support the maintenance of genomic integrity. Both BRCA1 and BRCA2 are involved in recognizing double-strand breaks (DSBs) and initiating the repair of damaged DNA through the homologous recombination (HR) repair system [38]. Thus, defective BRCAs lead to the accumulation of chromosomal breaks [39]. Furthermore, BRCAs serve as vital regulators of multiple transcription factors including p53. Germline mutations of *BRCA1/2* are frequently associated with somatic *TP53* abnormalities in patients with breast cancer [40]. Conditional mutants of *BRCA2* and *TP53* alleles predispose to mammary carcinogenesis [41, 42]. This is likely due, at least in part, to the ability of BRCA1 protein to interact with and regulate the transactivation of p53 target genes [43].

The accumulation of gene alterations in human cancers can give rise to the expression of tumor-specific neoantigens, which imply the potential of cancer cells to be recognized by the host immune system. In this regard, the tumor mutational burden (TMB) is a new biomarker to predict the potential of therapeutic strategies targeting tumor immunity. Tumors with germline or somatic mutations of BRCA1/2 are considered more immunogenic because of the dysregulation of the HR DNA repair system that causes increased genomic instability and high TMB. The existence of tumor-infiltrating lymphocytes in different breast cancer subtypes has been confirmed [44], with high frequency of infiltration of immune cells in breast cancers associated with *BRCA1* and *BRCA2* mutations [45, 46]. The immunotherapies targeting immune checkpoint receptors, such as PD1 and its ligand PD-L1 and CTLA-4, have emerged as a promising strategy to provoke an anti-tumor immune response in malignancies with polygenic mutations [47]. Indeed, many studies have proved the prevalence of PD-L1 expression in both breast cancer cell lines and clinical samples of breast cancer, mainly in TNBC patients [48–50], indicating that the aggressive subset of patients may benefit from PD1/PDL1 blockade. Of note, TNBC patients carrying the BRCA1/2-mutations display high TMB, suggesting that combined checkpoint blockade of PD-1 and CTLA4 might improve the efficacy of the chemotherapy treatment [46]. Breast cancers harboring *BRCA1* mutations are characterized by increased PD-L1 and PD-1expression, and a greater immune cells infiltration in the tumor microenvironment. Interestingly, these findings have not been observed in *BRCA2*-deficient tumors [51].

In this case report, we describe a TNBC patient who carries a *BRCA2* germline mutation and an array of somatic mutations, shows high expression of immune checkpoints and an elevated TMB with a dysregulated PI3K/AKT signaling pathway.

### Case presentation

**Fig. 1:**
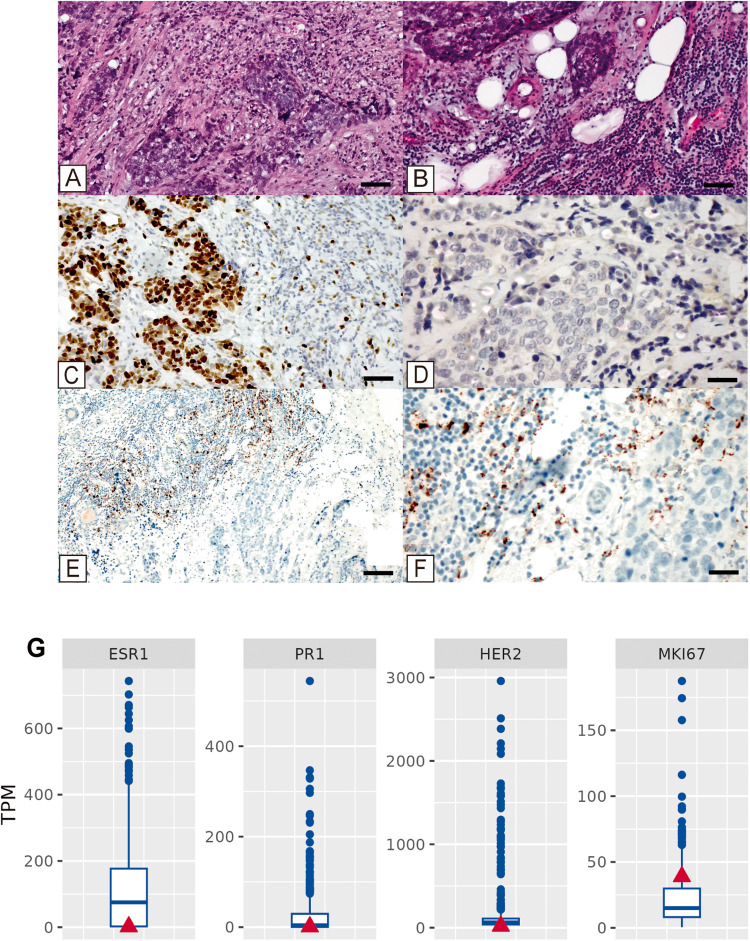
Histopathological analysis and molecular characterization of the tumor. **A** Hematoxylin and eosin staining shows a G3 infiltrating ductal carcinoma with inflammatory infiltrates. Scale bar represents 50 µm. **B** High magnification of panel A highlights the presence of peritumoral inflammatory cells (asterisk). Scale bar represents 50 µm. **C** Ki67 expression in more than 70% of breast cancer cells. Scale bar represents 50 µm. **D** c-Erb-B2 staining revealed score 0. Scale bar represents 20 µm. **E** PDL-1 (SP142) immunostaining shows positivity in more than 1% of tumor-associated lymphocytes (asterisk). Scale bar represents 100 µm. **F** High magnification of panel (**E**). scale bar represents 20 µm. **G** Expression mRNA levels (TPM) of estrogen receptor 1 (ESR1), progesterone receptor (PR1), ERBB2 receptor tyrosine kinase 2 (HER2) and proliferation marker KI-67 (MKI67) for the patient (red triangle) and the clinical cohort (blue boxplot).

In February 2021, an 81-year-old woman presented with a mass in the upper-outer quadrant of left breast. Histopathological examination revealed a G3 infiltrating ductal carcinoma with peritumoral lymphocytic inflammatory infiltrate (Fig. 1A, B); disease stage was IIA (pT2N0M0) (Table 1). Immunohistochemical (IHC) analysis showed strong positivity for Ki67 (~70%) (Fig. 1C), lack of expression of ER, PR, and HER2 (Fig. 1D). In addition, the IHC study of the expression of PD-L1 showed more than 1% of positive tumor-associated lymphocytes (Fig. 1E, F). All at once, IHC data allowed to classify the lesions as TNBC also suggesting a possible response to anti PDL-1 therapy. Lack of expression of ER, PR and HER2 was also confirmed by RNA-Seq, immunostaining and metabolic analyses [52–55]. As shown in Fig. 1G, the patient indeed showed absence of ER1, ER2, PR and HER2 mRNAs as compared to the clinical cohort (580 breast cancer patients). Further characterization of the tumor revealed a basal subtype of TNBC characterized by low to absent luminal differentiation marker expression, and high expression of epithelial-to-mesenchymal transition (EMT) and cancer stem cell-like markers (e.g., low claudin). The patient underwent a comprehensive genomic profiling that indicated the presence of four concurrent heterozygous somatic mutations in the ephrin type-A receptor 3 (*EphA3*), *TP53*, BRCA1-associated protein (*BAP1*) and *MYB* genes (Table 2).

**Table 1:** Clinical data of the breast cancer patient enrolled in this study.

Clinical information			
Gender	Female	Menarche	15 age of year
Age at case start	81	Menopause	Yes
General condition	Grade 0 Asymptomatic	Menopause at	34 age of year
		Reason	Natural
Weight	62.0 kg	Pregnancies	Yes (*n* = 4)
Body height	155 cm	Breastfeeding	Yes (*n* = 3)
BMI	25.8	Total month of breastfeeding	13
BSA	1.639		
Vegetarian	No	Hormone pre menopause	No
Meat consumption	2 times per week		
Defecation	Normal	Hormone post menopause	No
Smoker	No		
Disease ICD	Text		
C50.4	Malignant neoplasm of breast		
Tumor			
Organ	Breast		
Location	Upper-outer quadrant of left breast		
Histological type	Ductal carcinoma		
Morphology No	M-8500/3		
TNM	TNM Classification, 8^th^ Edition (UICC 2017) pT2 pN0(sn) 0/1 cM0 L0 R2		
Total radicality	R2		
Stage	IIA		
Grading	G3		
Dignity	Malign		
Ki-67	70		
Neoadjuvant therapy	No		
Subtype	ER- PR- HER2- /TNBC

**Table 2:** Genetic alterations detected in the tumor.

SOMATIC MUTATIONS				
Gene	Position	Original AA	Alteration	VAF
EPHA3	806	Asp	Asn	22%
TP53	214	His	Frameshift	29.80%
BAP1	51–53	–	Deletion	21.30%
MYB	603	Pro	Arg	14.50%

GERMLINE MUTATIONS				
Symbol	Feature ID	Effect	Nchange	AAChange
BRCA2	ENST00000544455.5	Splice donor variant & intron variant	c.516+1 G > C (intron variant)	p.Leu1839Ser

**Fig. 2:**
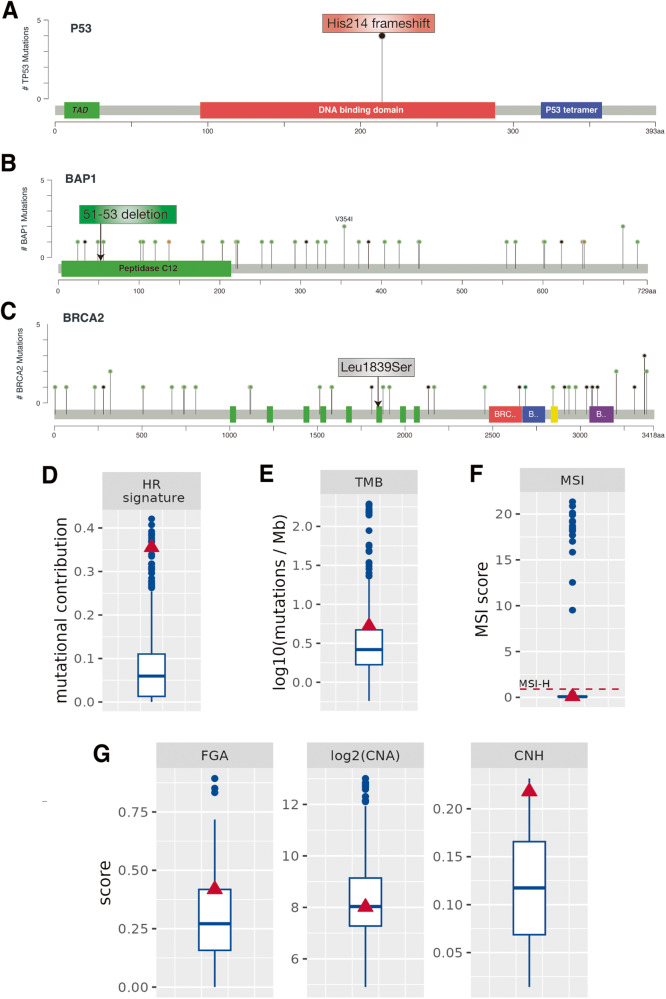
Molecular and chromosomal alterations in the breast cancer patient. **A** Schematic structure of the p53 protein and lollipop plot showing the incidence of mutations in the *TP53* gene in METABRIC cohort. Patient’s mutation is indicated. **B** Schematic structural features of the BAP1 protein and lollipop plot showing the incidence of *BAP1* gene mutations in METABRIC cohort. Patient’s mutation is indicated by arrow. **C** Schematic structural features of the BRCA2 protein and lollipop plot showing incidence of mutations in the *BRCA2* gene in METABRIC cohort. Patient’s mutation is indicated by arrow. **A**–**C** Data were obtained from cBioPortal. **D** Mutational contribution of HR-related signatures. **E** The patient has a higher TMB as compared to the cohort median (~80% percentile). **F** MSI score (MSI High: score > 0.901). The patient is observed as having MSI Low status (score = 0.09). **G** Chromosomal instability: CNH is higher in the patient compared to the median disease cohort, whereas numerical and structural CIN values are not much higher than median. The patient (red triangle) is compared to the clinical cohort (blue boxplot).

The His214 frameshift mutation of *TP53* (Table 2) lies within its DNA-binding domain (DBD), suggesting that it may impair its ability to contact DNA (Fig. 2A). This mutation has not been described previously in cancer patients. Interestingly, Yaupt and co-authors reported a truncated protein (p53d1214), composed by the first 214 amino-terminal residues of p53 that lacks the transactivation function, even though retains the ability to induce apoptosis [41, 56]. The relevance of this mutation in vivo and its biological significance warrants further studies.

We also identified a somatic mutation in the *BAP1* gene encoding a ubiquitin carboxy-terminal hydrolase that regulates several important cellular responses including HR DNA repair and cell growth [57, 58]. Germline inactivation of *BAP1* confers an increased risk for developing cutaneous and uveal melanoma, mesothelioma, renal cell carcinoma, and breast cancer, albeit to a lesser extent [59]. Mutations of *BAP1* have been included in the HR-deficiency-associated pathways in breast cancer, particularly in the TNBC subtype, characterized by a relative high mutation frequency [60]. In this patient we found the occurrence of a short deletion at amino acid 51–53 (Table 2), which lies within the peptidase domain of BAP1 and may likely affect its protein activity (Fig. 2B).

Genetic testing also revealed a germline variant (c.516+1 G > C; p.Leu1839Ser) located within the seventh repeat of the *BRCA2* gene (Table 2, Fig. 2C). This pathogenic variant harbors a G > A nucleotide substitution at position +1 of intron 6 of the *BRCA2* gene. The mutation, which eliminates a splice donor site, is predicted to alter RNA splicing. As a result, an abnormal mRNA could be produced, which could undergo to nonsense-mediated mRNA decay, or alternatively, an aberrant protein could be translated. This mutation has been reported in other breast and ovarian cancer patients [37].

Both the somatic mutations in the *TP53* and *BAP1* genes and the germline mutation in the *BRCA2* gene predict a HR-deficiency and chromosomal instability. Consistently, mutational signature analysis revealed a strong HR deficiency signature (Fig. 2D). We also observed a high tumor mutational burden (TMB) status in this patient as compared to the clinical cohort (Fig. 2E). The microsatellite instability (MSI) was instead stable as most breast cancer patients with the 0.1 score (Fig. 2F).

We also evaluated chromosomal instability (CIN) metrics, a principal indicator of aneuploidy and intra-tumor heterogeneity. We found that while the fraction genome altered (FGA, numerical CIN) and structural CIN (CAN) values of the patient are within the median range of the background cohort, her copy number heterogeneity (CNH) score is higher compared to the median disease cohort (Fig. 2G). The chromosomal instability represented by the high CNH value measured in the patient thoroughly correlates with tumor suppressor gene inactivation events [61] .

**Fig. 3:**
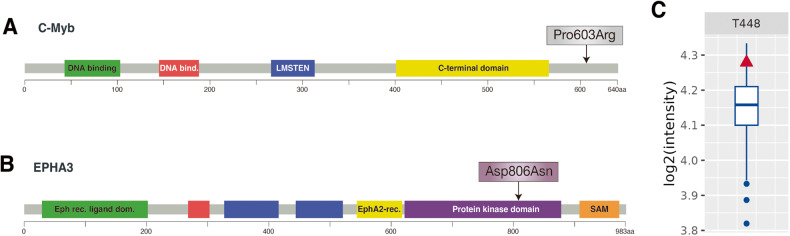
Genomic alterations in the patient. **A** *MYB* genomic alterations derived from the METABRIC dataset. Schematic structure of the c-Myb protein. Patient’s mutation is indicated by an arrow. **B** Schematic structural features of the EPHA3 protein. Patient’s mutation is indicated by an arrow. **A**, **B** Data were obtained from cBioPortal. **C** Increased AKT1 phosphorylation of T448 in the patient relative to the clinical cohort.

The patient also carries the Pro603Arg mutation in the *MYB* gene, which encodes the transcription factor proto-oncogene c-MYB. *MYB* is one of the prominent genes that gain frequent somatic copy number alterations in TNBC [62], while its mutations have never been reported in breast cancer patients and were not detected among the METABRIC patient cohort. Increased *MYB* expression confers resistance to tamoxifen in ER+ breast cancer cells by promoting EMT [63]. Therefore, deregulated expression of *MYB* could be used as an indicator to predict the response to drug therapy for breast cancer patients. The Pro603Arg mutation lies in the C-terminal domain whose function is currently unclear (Fig. 3A). Of note, since its deletion seems to increase the transcriptional transactivation activity of c-MYB, an inhibitory function has been proposed for the C-terminal domain of c-MYB [64].

The *ΕphA3* gene encodes a tyrosine kinase receptor, whose activation regulates important biological process altered in carcinogenesis, such as Rho-associated cell migration and adhesion [65]. Mutations and altered expression of *EphA3* have been associated with various human cancers, such as colorectal [66] and lung [67] carcinomas, in which *EphA3* dysfunction correlates with poor prognosis and decreased survival. Of note, EphA3 have available targeted treatment in phases I and II of clinical trials [68]. In particular, the anti-EphA3 monoclonal antibody KB004 has been explored in clinical trials aimed to treat refractory hematologic malignancies (NCT01211691) [69]. However, the role of EphA3 in breast cancer remains largely unexplored. An interesting study reported that EphA3 functions as a receptor for IL-26 in TNBC. IL-26 binding to EphA3 induces its dephosphorylation and dampens its activity, leading to increased phosphorylation of AKT and JNK and consequent tumor growth [70]. This patient harbors the Asp806Asn mutation (Table 2) that has also been described in colon cancer patients [66, 71]. The mutation is in the tyrosine kinase domain (Fig. 3B) and abolishes EphA3 tyrosine phosphorylation and, as a result, its enzymatic function [72]. It is therefore a an aminoacidic substitution that has a high potential to be pathogenic through AKT activation. In line with this possibility, we found the phosphorylation of threonine at position 448 of AKT1 protein is higher in the patient compared to cohort (Fig. 3C).

**Fig. 4:**
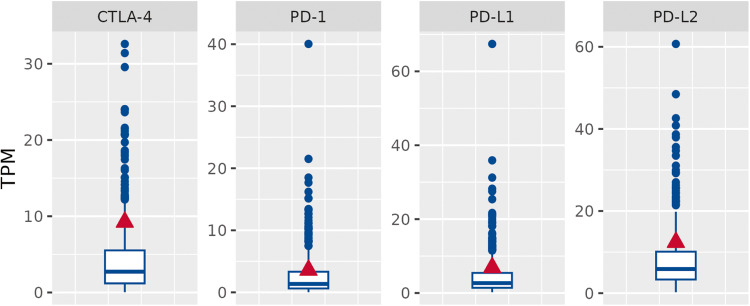
RNA-Seq expression levels of immune checkpoint genes in the patient. The patient (red triangle) is compared to the clinical cohort (blue boxplot).

Finally, evaluation of the immune checkpoints revealed high expression of PD-L1, PD-L2, PD1, and CTLA 4, relatively to the clinical cohort (Fig. 4). As reported above, the higher expression of PDL-1 (clone Sp142) was also demonstrated by IHC [73, 74].

## Discussion

An intact DNA damage repair response is fundamental to counteract cancer development. Defects in the HR repair system, occurring because of both somatic and inherited germline mutations in the HR pathway genes, can trigger deficient DNA damage responses and genome instability. In the case presented here, the patient had somatic mutations in the *TP53* (a frameshift mutation predicted to result in a transactivation deficient variant) and *BAP1* (a deletion variant that likely affects its deubiquitinase function) genes, and a germline mutation in the *BRCA2* gene, generating a risk pathogenic variant. We have coherently found mutational signatures associated with failures in HR pathways along with high TMB and CNH values. Since the dissatisfactory outcomes of conventional chemotherapies in TNBC patients, these features strongly indicate the importance to define the landscape of tumor immunogenicity, which would offer the basic principle to employ an optimal immune therapeutic strategy for cancer patients. There are few studies reporting the association of *BRCA2* mutations with immune biomarkers in tumors such as gastroesophageal cancers [75] or with an immunogenic microenvironment in ovarian cancers [76]. In contrast, the association of BRCA2 with immune checkpoints has never been reported in TNBC. The patient of our study showed higher expression of CTLA4, PD1, PDL1, PDL2 compared with the median expression of the clinical cohort. Hence, we have presented a clinical case that supports a link between *BRCA2* inactivation, HR deficiency and increased expression of immune checkpoints. Having a strong immunogenic signature, the patient might likely benefit from immunotherapies. HR deficiency status in TNBC may help identify suitable patients who would receive greater benefit from PARP inhibitors. *BRCA1/2* mutations can indeed help in predicting the sensitivity to poly (ADP ribose) polymerase (PARP) inhibitors. Clinical trials have also demonstrated a promising anticancer activity and safety of the combination of PARP and immune checkpoint inhibitors in patients with metastatic breast cancer harboring germline *BRCA1/2* mutations [77]. The clinical trial (NCT02657889) has proved that the anti-PD1 antibody combined with niraparib provided a favorable antitumor response in TNBC patients who carried tumor *BRCA* mutations [78]. Furthermore, the combined therapy of double immune checkpoint inhibitors (ICIs), such as anti-PD1 and anti-CTLA4, with chemotherapy provoked strong systemic and intratumoral immune responses in *BRCA1*-mutated breast cancer [46].

Interestingly, loss of *BAP1* expression was found to promote T cell infiltration in uveal melanoma [79]. Similarly, peritoneal mesothelioma and renal cell carcinoma patients with *BAP1* haploinsufficiency display an inflammatory microenvironment, characterized by increased immune cell tumor infiltration and enhanced PD-L1 expression [80, 81]. However, another study reported that loss of *BAP1* expression correlates with an immunosuppressive microenvironment in uveal melanoma, suggesting the immunotherapy resistance [82]. A patient affected by a BAP1 cancer syndrome, who developed a metastatic TNBC, carried both a germline pathogenic and a somatic *BAP1* mutation. Her tumor was PD-L1 positive, and the patient had a complete response to immunotherapy even after chemotherapy discontinuation. These findings indicate that *BAP1* alterations may also help predicting response to immunotherapy in breast cancer. This clinical case highlights the importance of screening TNBC patients for genetic mutations and TMB to predict the potential efficacy of immunotherapy.

Deregulation of the AKT pathway is a frequent event in breast cancer including TNBC. The PI3K/AKT signaling pathway is indeed commonly hyperactivated in TNBC as a result of AKT1 or PIK3CA mutations and/or PTEN inactivation [83]. To date, several AKT inhibitors have been tested in clinical trials. Some compounds proceeded to further development, being also tested in breast cancer patients in combination with chemotherapy, endocrine and anti-HER2 compounds [84]. Phosphorylated AKT levels are significantly associated with increased clinical benefit of the AKT inhibitor ipatasertib in TNBC patients [85], implying that the patient presented in this study would be probably sensitive to this treatment.

## Methods

### Collection of samples

Tumor tissues were globally collected using a standardized protocol, minimizing the ischemia time until freezing in liquid nitrogen. To ensure the quality of the samples, all tissues were Hematoxilin and Eosin stained and subjected to a pathological QC. Samples need to be invasive, have a tumor content of ≥30% and Necrosis ≤30%. Normal tissues were processed in parallel and need to be free of tumor and representative regarding the tumor tissue to be included.

Approximately 10 mg tissue were taken for nucleic acid extraction and protein lysate preparation each. To account for tumor heterogeneity, pathological QCs were performed on two sections, before and after taking the analysis material. The tissues stay frozen during the entire process.

### Immunohistochemical analysis

Approximately 1 × 1 × 0.5 cm of tissue was formalin-fixed and paraffin-embedded (FFPE). Serial sections were used to evaluate prognostic and predictive biomarkers including ER, PR, Ki67, and HER2 through immunohistochemistry. Briefly, sections were stained using the automated Leica Bond IHC platform (Leica Biosystems, Deer Park, IL). After antigen retrieval, 4-μm thick sections were incubated with the following primary monoclonal antibodies: mouse monoclonal anti-Ki67 (clone MM1; Leica Biosystems), mouse monoclonal anti-HER2 (clone CB11, Leica Biosystems) and rabbit monoclonal anti-PDL1 (clone sp142; Ventana Roche, USA). Reactions were revealed using BOND-PRIME Polymer DAB Detection System (Leica Biosystems, Deer Park, IL). Immunohistochemistry was evaluated by two blind pathologists.

### Nucleic acid extraction and quality assessment

Frozen tissue slices were mixed with beta-mercaptoethanol containing sample buffer and homogenized using the BeadBug system. DNA and RNA were extracted in parallel from the same sample using the Qiagen AllPrep Universal Kit according to the manufacturer’s instructions.

DNA and RNA concentration were quantified using Qubit fluorometer with the Qubit dsDNA BR assay or Qubit RNA BR assay respectively.

DNA and RNA quality were assessed using the Agilent Tapestation with the Agilent Genomic DNA kit or Agilent High-Sensitivity RNA ScreenTape kit respectively. RNAs need to have a RIN ≥ 4 or a DV200 ≥ 60 to be selected for library preparation.

### Library preparation and NGS sequencing

Libraries for whole genome sequencing (WGS) were prepared using the PCR-free KAPA Hyper Prep Kit (Roche). For whole transcriptome sequencing, RNA samples were depleted of the ribosomal RNA using the Ribo Zero Kit (Illumina) and library preparation was performed using the TruSeq Stranded Total RNA Kit (Qiagen). For small RNA sequencing the QIAseq miRNA Kit (Qiagen) was used All library preparation kits were used according to manufacturer’s instructions. Sequencing was performed on a NovaSeq6000 system (Illumina).

For WGS, average coverage for tumor samples was ≥60X and ≥30X for normal samples with a total genomic coverage of ≥95%.

Whole transcriptome sequencing datasets have ≥100 million total reads with <20% of ribosomal origin and ≥20 million reads mapping to mRNAs according to Ensembl reference. Ribosomal depletion was performed to remove nuclear rRNA and mt-rRNA.

### NGS data processing

NGS data was aligned against Grch38 genome assembly. Identification and annotation of short genomic variations in normal sample was done using Haplotype Caller (genome analysis toolkit; GATK) [86]. WGS somatic variation were called using a consensus of Mutect2(ref. 87), Strelka [88], Varscan [89] and Somatic Sniper [90]. Structural variations were called using R packages TitanCNA [91]and DellyCNV [92].

RNA-Seq differential expression was based on normalized readcount data (TPM: transcripts per million).

### Mass spectrometry phospho-proteome profiling

For phospho-proteome profiling, 5–10 mg of fresh-frozen tissue was lysed in 2 mL Precellys® CK14 tubes containing 1.4 mm ceramic beads and using a lysis buffer containing PhosSTOP™ and bead shaking using a Precellys® Evolution Homogenizer equipped with a Cryolys® cooling module. After overnight digest samples were acidified and subjected to peptide desalting using Waters HLB Oasis 30 mg 96-well plates. 500 μg of peptide preparation was subjected to phospho-peptide enrichment using MagReSyn® Ti-IMAC magnetic beads (ReSyn Biosciences) as described in ref. 93 with modifications to enable processing using a KingFisher™ Flex robot equipped with a 96-magnetic pin head. Peptides were desalted using Waters μElution plates, dried down and resolubilized.

For DIA LC-MS/MS measurements, 5 μg of peptides per sample were injected to a reversed phase column (nanoEase M/Z Peptide CSH C18 Column, 1.7 μm, 300 μm X 150 mm) on a Waters ACQUITY UPLC M-Class LC connected to a Thermo Scientific™ Orbitrap Q Exactive™ HF-X mass spectrometer equipped with an EASYspray source. The nonlinear LC gradient was 1–60 % solvent B in 60 min at 50 °C and a flow rate of 5 μL/min. The DIA method consisting of one full range MS1 scan and 50 DIA segments was adapted from Bruderer et al. [94].

Tissue-specific spectral libraries were generated combining high-fractionated DDA and DIA measurements on a pool of tissue material and raw data processed using Biognosys’ software Spectronaut 13.

### Bioinformatical analyses

Mutational signatures were calculated using the R package MutationalPatterns [95]. MSI classification was done using R package MSIseq [96]. PAM50 subtyping as well as risk scores were investigated using R package genefu [97].

TMB was calculated as the number of non-synonymous mutations of protein-coding genes divided by exome size in Megabases.
